# The added value of non-contrast 3-Tesla MRI for the pre-operative localization of hyperparathyroidism

**DOI:** 10.1016/j.bjorl.2021.07.010

**Published:** 2021-10-17

**Authors:** Yoshitaka Kawai, Mami Iima, Hirotaka Yamamoto, Makiko Kawai, Ayami Ohno Kishimoto, Sho Koyasu, Akira Yamamoto, Koichi Omori, Yo Kishimoto

**Affiliations:** a Kyoto University, Graduate School of Medicine, Department of Otolaryngology-Head and Neck Surgery, Kyoto, Japan; b Kyoto University, Graduate School of Medicine, Department of Diagnostic Imaging and Nuclear Medicine, Kyoto, Japan; c Kyoto University Hospital, Institute for Advancement of Clinical and Translational Science (iACT), Department of Clinical Innovative Medicine, Kyoto, Japan; d Tenri Hospital, Department of Otolaryngology, Tenri, Japan; e Department of Radiology, Kyoto City Hospital, Kyoto, Japan

**Keywords:** Primary hyperparathyroidism, Magnetic resonance imaging, Diagnostic imaging

## Abstract

**Objective:**

We investigated the efficacy of non-contrast 3-Tesla MR imaging added to the combination of sestamibi with^99m^Tc (MIBI) scintigraphy and Ultrasonography (US) for the pre-operative localization of Primary Hyperparathyroidism (PHPT) lesions.

**Methods:**

A total of 34 parathyroid glands, including nine normal glands, were examined with MIBI, US, and non-contrast 3-Tesla MRI. MRI was performed with the acquisition of T1- and T2-weighted images and fat-suppressed T2-weighted images. We calculated the sensitivities of MIBI, US, and the ‘additional’ MRI, with knowledge of the former two modalities’ results.

**Results:**

For the diagnosis of PHPT lesions, the sensitivity values of MIBI, US, and additional MRI were 88.0% (22/25), 84.0% (21/25), and 92.0% (23/25), respectively. Normal glands were not visualized with any modality (0/9). One lesion was detected neither with US nor MRI, but only with MIBI, with the limitation that MIBI represented no more than laterality. The two glands not identified in MRI were 4 mm and 6 mm in their size, which are within the range of normal gland’s size. Two lesions were not detected with US or MIBI but were visualized with the additional MRI, which indicated that the MRI contributed an 8.0% (2/25) improvement of sensitivity, compared from that of US. Fat-suppressed T2-weighted images were useful in the identification of parathyroid lesions, as these images helped to differentiate between the lesion and the adjacent tissue.

**Conclusion:**

Additional non-contrast 3-Tesla MRI was a useful adjunctive tool for localization of PHPT, which improved the sensitivity of the pre-operative localization of PHPT lesions. Fat-suppressed T2-weighted images contributed to their identification.

**Level VI:**

Evidence from a single descriptive or qualitative study.

## Highlights

• Sensitivity raised by unenhanced 3-Tesla MRI with US and MIBI in hyperparathyroidism.
• MRI could detect every parathyroid lesion that had been found in US.
• Undetected lesions in MRI were 6 mm or less, which are size of normal parathyroid.
• Fat-suppressed T2-weighted images could exhibit parathyroid lesions conspicuously.

## Introduction

Primary Hyperparathyroidism (PHPT) is caused by parathyroid lesions. Approximately 90% of the cases of PHPT is caused by a single parathyroid adenoma, 5% by multiple gland hyperplasia, 4% by double adenomas, and 1% by parathyroid carcinoma.^1^ For the treatment of PHPT, surgery is the only road to definitive recovery. As the parathyroid gland is small and its location varies among patients, a precise pre-operative assessment should be conducted for a successful surgery. For the routine surveillance of PHPT patients, the combination of Ultrasonography (US) and ^99m^Tc sestamibi (MIBI) scintigraphy is widely used.^2^ With US, detailed images of organ structures can be visualized, and the blood flow of the scanning area can be observed in real-time. As the parathyroid gland is characterized by its abundant blood supply, the evaluation of suspected nodule's circulation with the Doppler mode of US is very informative.

MIBI scan is a radioisotope scintigraphy that was originally developed to measure the blood perfusion of the heart, which was eventually demonstrated to be useful for scanning the parathyroid. Compared to conventional ^201^Tl-^99m^TC subtraction imaging, the images acquired by MIBI have better contrast with a lower radiation dose.^3^ Compared to reciprocal planer imaging, a recently developed method using Single Photon Emission Computed Tomography and conventional Computed Tomography (SPECT/CT) fusion provides better localization of the tracer.

However, these experiments have their own limitations. The scanning area of US is relatively small, which makes it challenging to share the detailed information regarding lesions and anatomical structure with medical staff before surgery. Sometimes comparing US findings with MIBI imaging is difficult, due to the inability of recording the scanning angle of US. Additionally, the sensitivity of US highly depends on the skill of the examiner. The limitations of US also include the inability to identify ectopic adenomas in the mediastinum.^4^ Regarding limitations of MIBI imaging, it is unavailable for patients who are pregnant due to the risk of radiation exposure.

Magnetic Resonance Imaging (MRI) is a noninvasive diagnostic modality which can produce detailed images of the whole neck without using any radiation. It has been applied in the localization of PHPT, though that is not commonly performed. With the improvement from the 1.5-Tesla magnet to 3-Tesla, one could expect better signal-to-noise and the contrast-to-noise ratios, leading to a better detection of small lesions.^1^ In addition to commonly acquired axial T1- and T2-weighted sequences,^2^, ^4^, ^5^ the acquisition of gadolinium-enhanced sequences is expected to increase the detectability of lesions.^6^ However, the use of gadolinium contrast agents requires careful consideration because of gadolinium’s retention and toxic characteristics in brain and other tissues, and the risk of developing Nephrogenic Systemic Fibrosis (NSF) in patients with renal impairment.^7^

Several articles inferred the possibility of fat-suppression sequences without contrast reagent, for the localization of PHPT.^8^, ^9^ These sequences were extensively developed in the 1990s and described articles are based mainly on 1.5-Tesla MRI, and few studies have investigated the utility of non-contrast 3-Tesla MRI in the pre-operative localization of PHPT.

Here, we investigated the complementary diagnostic performance of non-contrast 3-Tesla MRI in combination with US and MIBI for the detection of parathyroid lesions. Note that the study patients’ MRI findings were evaluated after their US and MIBI findings were obtained, with knowledge of all of those results, as our aim was to evaluate the utility of MRI as an adjunctive tool to other modalities.

## Patients and methods

### Patients

This retrospective study protocol was approved by the ethical committee of our institute. Between December 2015 and January 2020, patients with a clinical diagnosis of PHPT who underwent a pre-operative MRI examination at our institute were enrolled. Fine-needle aspiration cytology was not performed in any case. We excluded patients not taking MIBI scintigraphy or US, patients with skipped essential MRI sequences described in the following section, and hemodialyzed patients who may have had secondary hyperplasia.

All patients underwent surgery, and all resected parathyroid glands were histopathologically confirmed. The surgically resected parathyroid glands were documented and anatomically labeled in a standard location pattern by a certified otolaryngologist, which allowed us to correlate the pathological and radiological results. To minimize the stress of surgical procedure, unilateral approach was basically applied, except one patient who had a concomitant thyroid carcinoma. Intact Parathyroid Hormone (iPTH) levels both pre- and post-surgery were measured and recorded in all patients.

### Study protocol

#### MIBI scintigraphy

Based on our hospital’s protocol, SPECT/CT was performed using a SPECT/CT scanner (Infinia Hawkeye 4®, GE Healthcare, Waukesha, WI, USA; this has a dual-head gamma camera) and integrated low-dose, four-slice CT. Each patient was intravenously injected with approx. 600 MBq of ^99m^Tc-MIBI. Anterior planar images from the neck to the thorax were acquired at 15 min (early images) and at 2–3 h (delayed images) after the injection. After the acquisition of delayed planar images, SPECT/CT images were acquired from the neck to the thorax. The emission images were superimposed with the patient’s CT data to generate hybrid images.

The sensitivity of MIBI scintigraphy was based on each diagnostic report written by a board-certificated nuclear medicine physician. The results that indicated only laterality without superior-to-inferior locations were considered positive.

#### US

Each US examination was performed using a Xario 100 or Xario XG system (Toshiba Medical Systems; Tokyo). A real-time scanner with a 7.5 MHz linear probe (Toshiba Medical Systems) was used to identify abnormal parathyroid glands. A careful examination of the parathyroid gland, thyroid gland, and surrounding tissues of the neck was performed. The sensitivity of the US was based on each diagnostic report written by a board-certificated radiologist or a head and neck surgeon.

#### Additional MRI

All patients were examined with non-contrast 3-Tesla MRI (Prisma or Skyra, Siemens Healthcare, Erlangen, Germany). The following images were acquired: a T1-weighted image (T1WI; TR/TE, 600/12), a T2-Weighted image (T2WI; TR/TE, 2000/102), and a fat-suppressed T2-weighted image (STIR; Short TI Inversion Recovery or SPAIR; SPectral Attenuated Inversion Recovery). The sensitivity of ‘additional’ was based on the review by the author, who was blinded to the histology result but aware of the US and MIBI scintigraphy data.

### Data analysis

We calculated the sensitivity, specificity, Positive Predictive Value (PPV), Negative Predictive Value (NPV), and accuracy.

We divided all the parathyroid glands into three groups based on the histopathological diagnosis: adenoma, hyperplasia, and normal gland. The pathological differential diagnosis between adenoma and hyperplasia was made depending on whether there was a single abnormally proliferating gland or multiple abnormally proliferating glands. Parathyroid hyperplasia was originally reported as a disease of all four parathyroid glands,^10^ but the differentiation between multiple adenomas and hyperplasia is difficult because of their similar histological features and the existence of ‘focal’ hyperplasia, which represents unequally enlarging glands in a single patient.^10^

The parathyroid glands were also classified according to each gland’s maximum diameter: ≥10 mm, <10 mm, or unknown. We set the cut-off as 10 mm because a previous investigation indicated that ≥10 mm lesions could be easily localized with US.^11^ The diameter was confirmed by the pathological examination reports or surgery records. Some samples lacked a record of their diameter and were classified as ‘unknown’.

## Results

The investigated samples were 34 histopathologically confirmed parathyroid glands resected from 23 patients with a clinical diagnosis of PHPT (7 males and 16 females; aged 37–80 yrs.; median age 58 yrs.). In the pathological diagnosis, 21 glands (21 patients) were adenoma, four glands (two patients) were hyperplasia, and the remaining nine glands were normal and unremarkable parathyroid glands. Before surgery, all patients exhibited elevated iPTH values ranging from 85 to 718 pg/mL (median 171 pg/mL; normal range 10–65 pg/mL). The post-operative iPTH values ranged from 3 to 89 pg/mL (median 32 pg/mL). The decreased iPTH values after surgery were observed in all patients, except one unrecovered hyperplasia case.

### Sensitivity of each modality

For the detection of parathyroid gland lesions, the sensitivities of MIBI, US, and additional MRI (combined with MIBI and US) were 88.0% (22/25) for MIBI, 84.0% (21/25) for US, and 92.0% (23/25) with the additional MRI. An adenoma represented a tricky pattern, which was detected with MIBI, but neither in US nor in MRI. In this case, laterality of the lesion was displayed with planar image of MIBI, however, its precise location was not well exhibited in SPECT image of MIBI, or other modalities.

The statistical values of these modalities were presented in Table 1. The sensitivity of each subgroup arranged by histopathological groups and gland diameters are summarized in Table 2.

**Table 1:** The diagnostic performance of each modality.

	Sensitivity (%)	Specificity (%)	PPV (%)	NPV (%)	Accuracy (%)
MIBI	88.0 (68.8–97.5)	100 (66.4–100)	100 (N/A)	75 (50.9–89.7)	91.2 (76.3–98.1)
US	84.0 (63.9–95.5)	100 (66.4–100)	100 (N/A)	69.2 (47.8–84.7)	88.2 (72.6–96.7)
MRI after MIBI and US	92.0 (74.0–99.0)	100 (66.4–100)	100 (N/A)	81.8 (54.4–94.5)	94.1 (80.3–99.3)

Numbers in parentheses indicate 95% Confidence Interval.

**Table 2:** Number of detected glands at each modality according to lesion characteristics and diameter.

Pathology	Gland diameter	Nº of glands examined	MIBI	US	MRI after MIBI and US
Adenoma	Total	21	19 (90.5%)	18 (85.7%)	20 (95.2%)
	≥10 mm	18	17 (94.4%)	17 (94.4%)	18 (100%)
	<10mm	3	2 (66.7%)	1 (33.3%)	2 (66.7%)
	Unknown	0	–	–	–
Hyperplasia	Total	4	3 (75.0%)	3 (75.0%)	3 (75.0%)
	≥10 mm	3	3 (100%)	3 (100%)	3 (100%)
	<10mm	1	0 (0%)	0 (0%)	0 (0%)
	Unknown	0	–	–	–
Adenoma + Hyperplasia	Total	25	22 (88.0%)	21 (84.0%)	23 (92.0%)
	≥10 mm	21	20 (95.2%)	20 (95.2%)	21 (100%)
	<10mm	4	2 (50.0%)	1 (25.0%)	2 (50.0%)
	Unknown	0	–	–	–
Normal	Total	9	0	0	0
	≥10 mm	0	–	–	–
	<10mm	3	0	0	0
	unknown	6	0	0	0

Numbers in parentheses indicate sensitivities in the subgroup.

Normal parathyroid glands were not detected by any modality, probably due to their small size. This resulted in the 100% PPV in all modalities. For the detection of ≥10 mm enlarged parathyroid glands, the sensitivity of MRI was 100% (22/22). In the group of <10 mm parathyroid gland lesions, the sensitivity of MRI was 50% (2/4). The detected adenomas were 8 mm and 9 mm, and the undetected adenoma and hyperplasia gland were 4 mm and 6 mm in size.

### Diagnostic value of fat suppressed T2WI

**Figure 1:**
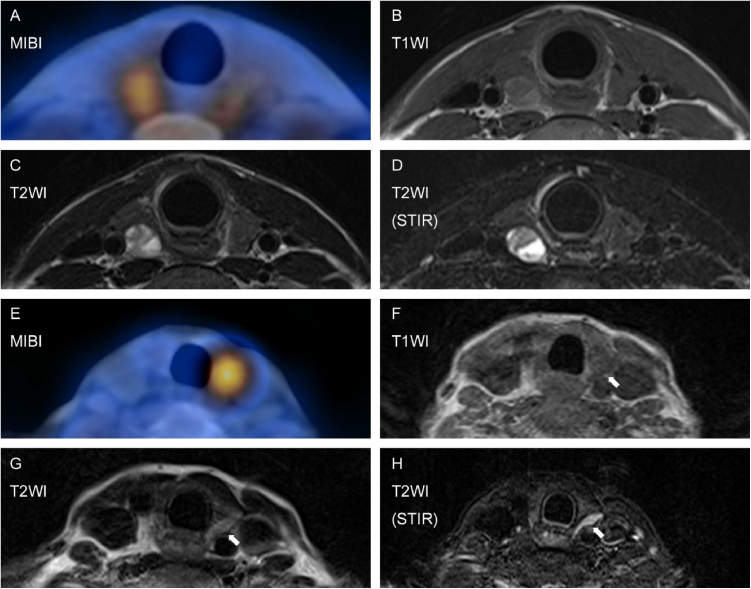
Images of typical PHPT (A–D) and ‘flattened’ bean-shaped PHPT (E–H). Delayed phase of MIBI (A). The lesion represented iso signal intensity (SI) on T1WI (B), iso-high SI on T2WI (C), and stronger SI on fat-suppressed T2WI (STIR: D). In a case of an atypically shaped adenoma, the lesion indicated by MIBI (E) presented equivocal-iso SI on T1WI (F) and T2WI (G). The lesion conspicuity increased on fat suppressed T2WI (H).

The typical appearance of a parathyroid gland is low-to-medium Signal Intensity (SI) on T1WI, medium-high SI on T2WI, and stronger SI on fat-suppressed T2WI (Fig. 1). Fat-suppressed T2WI is thought to contribute substantially to lesion detection, when the shape of a PHPT lesion is atypical ‘flattened’ bean shape rather than ordinary round shape. With the help of fat suppressed T2WI, all PHPT lesions localized with US were also detected with MRI.

### Details of cases in which MRI played crucial role for localization

**Figure 2:**
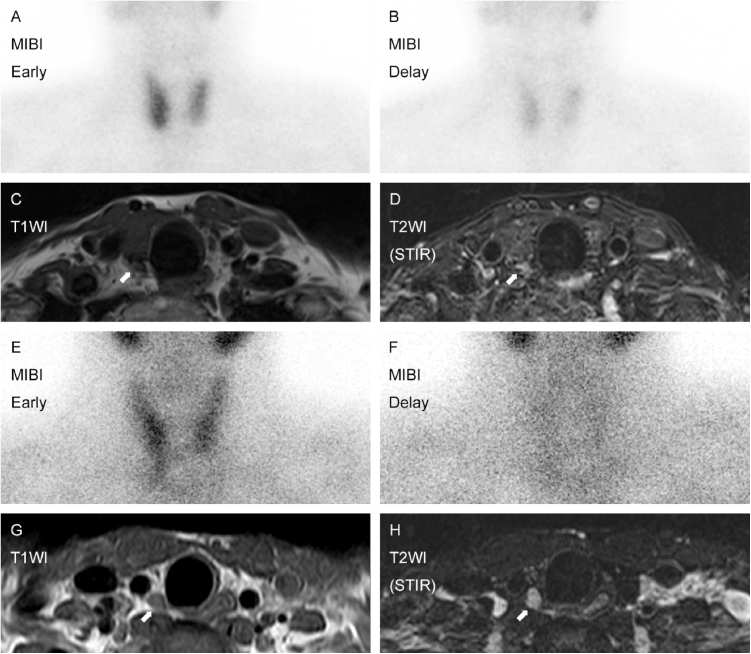
Two cases detected by MRI which were not initially seen on US. Initial case was a 76-year-old female with 8 mm adenoma. MIBI presented diffuse uptake on the right side (A and B). Not US but MRI revealed the lesion (C and D). Another case was a 35-year-old male with 17 mm adenoma. MIBI represented equivocal accumulation on the right inferior quadrant (E and F). The lesion was successfully detected by MRI (G and H), which were not initially localized on US.

In this study, two adenomas were localized after MRI examination, but they were not well identified at the time MIBI, and US were finished. Their MIBI and MR images are shown (Fig. 2). A minimally invasive single gland resection could thus be performed in these cases, followed by successful iPTH reduction.

## Discussion

Although MIBI and US are the most commonly used imaging modalities for the pre-operative examination of individuals with PHPT, their sensitivities differ largely among the prior studies. According to a review, a recent large series examined by MIBI with SPECT showed sensitivities in the range of 68%–95%, and the sensitivity calculated in a meta-analysis of 96 studies of solitary adenoma was 88%; the sensitivity for hyperplasia was 44%.^2^ The same review also reported the sensitivities of US, which ranged from 72% to 89% in large series, and a meta-analysis reported that the sensitivity values of US for solitary adenoma and hyperplasia were 79% and 35%, respectively.^2^ The sensitivities of MIBI and US in the present study show only a slight degree of divergence from these reports.

Several reports have described the sensitivity of MRI for the detection of parathyroid lesions; 1.5-Tesla MRI has shown unsatisfactory sensitivity at 43%–77%, due to its limited spatial resolution and motion artifacts.^4^, ^12^ At present, better sensitivity have been obtained with contrast-enhanced 3-Tesla MRI examinations. In studies of four-Dimensional (4D) MRI, which tracks the time-resolved enhancement kinetics of lesions, 85%–90.5% sensitivity was reported.^13^, ^14^, ^15^ An investigation of non-contrast 3-Tesla MRI demonstrated 81.0% sensitivity.^15^

Our present finding of 92.0% sensitivity for parathyroid lesions (23 of 25 lesions) is from a mixture of MIBI, US, and MRI rather than a single modality. The additional contribution of MRI might be considered an 8.0% (2/25) improvement of sensitivity, compared from that of US (21/25). In appearance, MIBI also represented a good sensitivity (22/25), however, the positives of MIBI include ones which represented only laterality but not precise location of the lesions. It would be tough to say laterality and accurate spot have same values for a surgeon. A detailed description of the lesion sizes might explain the reason for false negatives of MRI; the diameters of the detected adenomas were 8 mm and 9 mm whereas those of the undetected adenoma and hyperplasia gland were 4 mm and 6 mm. Considering normal parathyroid gland measures approximately 6 mm,^16^ the glands not identified in MRI were not enlarged. Our results indicate that MR images are not always helpful in cases of smaller lesions, due to its inability to visualize their function.

Represented specificity, NPV, and accuracy in this study might be unreliable, as normal glands were basically unintendedly resected, not in accordance with a rule. The calculated performance from such data might not reflect the actual capacity of each modality, due to the obviously small sample number of normal glands.

The efficacy of a fat-suppressed sequence in PHPT localization has not been well established. Some scholars have investigated the utility of a fat suppressed sequence with a contrast agent, named IDEAL (Iterative Decomposition of water and fat with Echo Asymmetry and Least-squares estimation), reporting 95.7%–97.8% sensitivity for detecting parathyroid lesions.^1^, ^12^ Another research group reported the bright hyperintensity of non-contrast fat-suppressed T2WI, but its contribution to the localization of lesions was not well documented.^17^

In our study, the contribution of fat suppressed T2WI resulted in the 100% sensitivity of MRI for detecting PHPT lesions that were pointed out with US. Even considering that the MRI scan was performed after the US for each patient, this high sensitivity encourages the use of MRI for pre-operative patients, so that the anatomical location of the detected lesion can be shared with the medical staff.

As a second line of parathyroid lesion detection, CT is also commonly used. To improve the sensitivity of CT, a 4D-CT technique was recently applied for PHPT lesions; for the detection of single-gland disease, its sensitivity is 92%–94%, but the sensitivity drops to 44%–59% for the detection of multi-gland lesions.^18^, ^19^ Pitfalls of 4D-CT are the requirement of an experienced reader and the high radiation dose to the thyroid. The radiation dose of 4D-CT is 13–50 times higher than that of nuclear scintigraphy.^20^, ^21^ Considering that radiation exposure in younger patients would result in an increased cancer risk, the use of 4D-CT for young patients requires prudent deliberation.

We are aware of the considerable limitations in this study. First, the sensitivity of MRI in this study is strongly affected by confirmation bias; MRI was reviewed with the knowledge of US and MIBI results, which might lead to recognize an ambiguous nodule in MRI as the target lesion, already found in other modalities. However, we don’t take this seriously. Even if MRI finding of a PHPT lesion remains to be an uncertain nodule, we can ‘clinically’ predict that nodule as the most likely location of the lesion, as long as US and MIBI represented consistent findings. Second, the present study design is a descriptive study without any comparison, rather than a cross sectional study comparing among modalities. As MRI was not reviewed in blind, the better sensitivity of MRI would not mean to beat those of US or MIBI. Additionally, this descriptive study would not tell the relation between the MRI findings and clinical outcomes, i.e., operation time, amount of frozen section procedure, post-operative iPTH amount, and recurrence ratio. Lastly, the smallness of sample size directly affects to the reliability and reproductivity of results. Present study is a single institution study and remains to be a preliminary data.

In conclusion, our results indicate that non-contrast 3-Tesla MR imaging is helpful as a complementary imaging modality for the localization of PHPT lesions, improving the sensitivity in combination with MIBI scintigraphy and US. In the transcription of MR, the acquisition of fat suppressed T2WI contributed significantly to the localization of PHPT lesions. All lesions detected with US could be confirmed with MRI, which infers that MRI examination for PHPT patients enables sharing the anatomical location of identified lesion with medical staff, resulting in better localization and more successful surgical treatment of PHPT lesions.

## Conflicts of interest

The authors declare no conflicts of interest.
